# The effect of unemployment and post-natal care on the exclusive breast-feeding practice of women in Ethiopia: a systematic review and meta-analysis

**DOI:** 10.1186/s12978-022-01404-y

**Published:** 2022-04-15

**Authors:** Mekdes Hailegebreal Habte, Seada Jemal Seid, Ayinalem Alemu, Hanna Abera Hailemariam, Birhanu Asrat Wudneh, Rahel Nega Kasa, Zebenay Workneh Bitew

**Affiliations:** 1 St. Paul’s Hospital Millennium Medical College, Addis Ababa, Ethiopia; 2 Ethiopian Public Health Institute, Addis Ababa, Ethiopia

**Keywords:** Exclusive breast feeding, EBF, Post natal care utilization, Maternal employment status, Ethiopia

## Abstract

**Background:**

Promoting exclusive breastfeeding (EBF) is a major child survival strategy in developing countries like Ethiopia. Studies in EBF are found in a fragmented and inconclusive way in Ethiopia. Therefore, the aim of this study was to examine evidences on the effect of post natal care counseling and maternal employment status on exclusive breastfeeding practice of women in Ethiopia.

**Methods:**

A systematic literature search was conducted from PubMed (contains MEDLINE), CINAHL (EBSCO), Global Health, Food Science and Technology Abstracts (FSTA) (EBSCO) and Grey literature sources such as Google and Google scholar. All primary studies on the effects of employment status and/or post-natal care utilization on EBF practices of women in Ethiopia were included. Data analyses were performed using STATA software. Forest plot, I^2^ test and the Cochrane Q statistics were used to detect heterogeneity among studies. Heterogeneity was considered significant when the I^2^ value was ≥ 50%, with p-value < 0.05. Publication bias was checked by looking the asymmetry of funnel and confirmed by Egger’s regression test at a 5% significant level. The pooled odds ratio (POR) with 95% confidence interval (CI) was used to report the measures of associations.

**Result:**

A total of 622 studies were identified in the initial search of which 42 articles were included this systematic review and meta-analysis. A meta-analysis of 24 studies indicated that maternal employment status was significantly associated (POR = 0.51, 95% CI 0.16, 0.86) EBF practice in that employed mother were less likely to practice to practice EBF. Post-natal care service utilization significantly increases (POR = 1.76, 95% CI 1.32, 2.34) the EBF practice in Ethiopia and it was computed using 25 eligible articles. Besides, the pooled estimates of EBF practice was found to be 62.58% (95% CI 56.98, 68.19, I^2^ = 96.4%, p < 0.001).

**Conclusion:**

This review found that post-natal care service utilization and maternal employment status has a significant effect on EBF practice. The findings from this review may be used to inform for better supportive and promotive strategies for EBF practice in Ethiopia.

**Supplementary Information:**

The online version contains supplementary material available at 10.1186/s12978-022-01404-y.

## Plain Language summary

Promotion of EBF in developing countries including Ethiopia is very crucial strategy to improve the survival of children. Despite this, several factors prohibit women from practicing EBF of which post-natal care service utilization and employment status are the main determinants. The previous studies in Ethiopia are inconsistent and inconclusive to policy makers. Thus, this systematic review and meta-analysis underpinned the effects post-natal care service and maternal employment status on the EBF practice of women in Ethiopia. The findings of this study will be vital to forward recommendations on the duration of maternal leave and design promotion strategies of post-natal care, the most unaddressed maternal health care service in Ethiopia. The original studies were retrieved systematically from reputable databases and grey literature sources. The data were extracted using standardized data extraction sheet. Analyses were performed using STATA software. Fixed and random effect models were utilized to compute pooled estimates. Heterogeneities were computed and the sources of heterogeneities were explained. In the current study, the pooled prevalence of EBF was 62.6%. Likewise, post-natal care service utilization and employment status were found to have significant association with the EBF practice. Unemployed women had a better EBF practice as compared to the employed ones. This could be due to the fact that most employed women in Ethiopia are governmental employees where the maximum duration of maternal leave is four months after birth. This is before the due date of commencement of complementary feeding. Post-natal care should also be promoted to improve the EBF practice.

**Supplementary Information:**

The online version contains supplementary material available at 10.1186/s12978-022-01404-y.

## Introduction

Exclusive breastfeeding (EBF) is globally promoted as the ideal method of infant feeding during the first 6 months of life due to its health benefits to both the mother and the child [1]. Exclusive breastfeeding is an ideal way of achieving optimal child growth and improves the brain development of the child and intelligence [2]. Exclusive breastfeeding is noted as a major child survival strategy. Currently, the global prevalence of EBF for infants aged 0–6 months is only 38% which is far behind to make EBF during the first 6 months of life the norm for infant feeding indicates that 11.6% of mortality in children under-5 years of age was contributed by non-exclusive breastfeeding [3].

Sustainable Development Goal (SDG) targets for child mortality represents a renewed commitment to the world’s children: by 2030. Preventable deaths of newborns and children’s under 5 years of age, in all countries aim to reduce neonatal mortality to at least as low as 12 deaths per 1,000 live births and under five mortality to at least as low as 25 deaths per 1000 live births. However, a child dies in every 5 s globally, approximately 18,000 children every day, and if current trend continues with more than 50 countries falling short of the SDG target on child survival, some 60 million children under age 5 will die between 2017 and 2030 and half of them will be newborns [4, 5].

Increasing breastfeeding is identified as a priority area supported by current policy targets, with global targets recently increased 50% of children being exclusively breastfed at 6 months by 2025 to at least 70% by 2030 [6]. Despite the presence of high impact interventions to improve infant and young child feeding, only about 52% of mothers in Ethiopia, exclusively breastfeed their child for the first 6 months after delivery [7].

In Ethiopia, different studies have been conducted to determine factors associated with EBF. These studies showed that maternal and health service-related factors including, giving birth in health facilities, being housewife in occupation, receiving counseling/advice on infant and colostrum’s feeding, and maternal employment status influenced the practice of exclusive breast-feeding [8–11]. Among these factors, post-natal counseling is an effective public health intervention to increase rates of exclusive breast feeding up to 6 months post-partum. Due to urbanization and levels of education, the proportion of employed women in Ethiopia has been increasing gradually [12]. To attain progress on the global exclusive breastfeeding target by 2025 women should be empowered to practice EBF by providing 6 months of mandatory paid maternity leave and countries are expected to make policies that create a conducive environment for breastfeeding in the workplace and help women to breastfeed their children exclusively for the first 6 months and thereafter [13, 14]. Despite its demonstrated benefits, exclusive breast-feeding practice in many countries including Ethiopia is lower than the international recommendation. As far as our knowledge is concerned, though there were several studies with inconsistent results, there is no published systematic review and meta-analysis in Ethiopia, which investigated the effect of post-natal care service utilization and maternal employment on EBF practice of women. Therefore, the purpose of this systemic review and meta-analysis was to examine evidence on the effect of postnatal care service utilization and maternal employment status on exclusive breastfeeding practice of women in Ethiopia. Findings from this review will provide evidentiary inputs for policy makers, managers and decision makers to improve EBF practice in Ethiopia.

## Research methods

### Searching strategies and data sources

The present systematic review and meta-analysis is performed with the intent of determining the effects of employment status and post-natal care service utilization on the EBF practices of women in Ethiopia. The findings of the current study were arranged based on the Preferred Reporting Items for Systematic Reviews and Meta-analysis (PRISMA) guideline [15]. Articles published from the date the research inception to November 2021 were searched from different databases. Comprehensive searching was conducted from the databases: PubMed (contains MEDLINE), CINAHL (EBSCO), Global Health, Food Science and Technology Abstracts (FSTA) (EBSCO). Grey literature sources such as Google and Google scholar were also explored thoroughly to retrieve articles. The reference lists of included studies were also crosschecked to find out additional articles. Articles identified through the electronic search were exported and managed using EndNote X8 software. The Boolean search operators such as “OR”, “AND” were used during the searching process. Key terms were verified for appropriateness prior to the actual searching. The following search string was employed to retrieve articles from PubMed:

(("Breast Feeding"[MeSH Terms] AND (("associate"[All Fields] OR "associated"[All Fields] OR "associates"[All Fields] OR "associating"[All Fields] OR "association"[MeSH Terms] OR "association"[All Fields] OR "associations"[All Fields]) AND ("factor"[All Fields] OR "factor s"[All Fields] OR "factors"[All Fields]))) OR (("maternally"[All Fields] OR "maternities"[All Fields] OR "maternity"[All Fields] OR "mothers"[MeSH Terms] OR "mothers"[All Fields] OR "maternal"[All Fields]) AND ("employability"[All Fields] OR "employable"[All Fields] OR "employer"[All Fields] OR "employer s"[All Fields] OR "employers"[All Fields] OR "employment"[MeSH Terms] OR "employment"[All Fields] OR "employments"[All Fields])) OR " post-natal Care"[MeSH Terms]) AND "Ethiopia"[MeSH Terms].

Finally, the PICO was followed to in the searching process:

- Participants/population: Mother infant pairs.
- Intervention(s)/exposure(s) group: Employed women and women who attend post-natal care services.
- Comparator group: unemployed women and women those who didn’t attend post-natal care.
- Outcomes of interests: exclusive breast feeding practice of women in Ethiopia.

### Inclusion and exclusion criteria

The study selection process was conducted by three authors (ZWB, SJS and MHH). Studies were assessed using study area, study design, title, and abstract. Finally, assessment of the full texts was performed based on the pre-defined inclusion criteria. All observational studies (cross-sectional and case–control) that reported the effects of employment status and post-natal care utilization on the EBF practice of women were included in this study. Studies reporting the association using odds ratio were included and the Cruds odd ratios were used in this meta-analysis. Systematic reviews and meta-analyses, non-human studies, studies not reporting the outcome of interests, conference proceedings, qualitative studies, case reports, editorial comments, and studies conducted other than English language were excluded from the current study. Besides, studies that did not report odds ratio based on two by two tables were also excluded due the fact we are unable to identify the original data.

### Data extraction process

Data extraction was conducted by three authors (ZWB, MHH, BAW and HAH) using a standardized data extraction form. Microsoft excel, 2016 was used to sort out the data by two authors independently. Then, the data was cleaned and get ready for the final analysis using the excel spreadsheet. Lastly, the data were exported to the STATA software (Version 16) for the final analysis. The data extraction format included: name of the author(s), publication year, study region, study population, study design, sample sizes, EBF practice, and factors. Discrepancies between the authors were solved through discussion and consensus, and with active involvement of the other authors (SJS and RNK) (Additional file 1).

### Quality assessment of studies

Quality assessment was performed by two authors (ZWB and MHH) independently using a Joanna Briggs Institute (JBI) Critical Appraisal Checklist for Observational Studies [16]. The tool contains options: Yes, No, Unknown, and Not Applicable. One is given for yes and zero for all other options. Finally, the scores were summed up and changed to percentages. Studies with a quality score of > 50% were included in this meta-analysis (Additional file 2). The mean scores of the two reviewers were used for final decision of inclusion of the studies in this systematic review and meta-analysis. The inter-rater agreement was computed by an author (AA) using Cohen’s kappa coefficient (κ). The findings show substantial agreement between the two raters (κ = 0.68, p < 0.001). Finally, studies were included accordingly.

### Outcome measures

The effect of employment status on the EBF practice of women was the primary outcome of the current study. The second outcome was the effect of post-natal care service utilization on the EBF practice of women in Ethiopia. Odds ratio was used to identify the effects employment status and post-natal care service utilization on the EBF practice of women. The pooled estimates for two outcomes were computed as follows. The individual components of the factors were computed first as follows: (a) the number of EBF practice among the factors (employed or those who attend postnatal care); (b) the number of non EBF practice among the factors; (c) the number of women who were not employed and practiced EBF; and (d) the number of women who were not employed and not practiced EBF. Then, the crude odds ratios for the individual studies were computed (OR = ad/bc). Finally, the pooled odds ratios for the two outcomes were computed accordingly. The “metan” command was used to compute the pooled estimates using STATA (version 16) software. Lastly, the pooled estimates were presented with their 95% CIs. The effect sizes were odds ratios indicating the association between EBF practice of women and employment status of women or post-natal care service utilization.

### Statistical methods and analysis

In this study, STATA Version 16 (STATA Corporation, College Station Texas) software was used to compute the pooled odds ratios. Both random and fixed effect models were employed to compute pooled estimates. However, a random-effect model weighted using the inverse variance method was used to report the final pooled estimates due to the presence of high heterogeneity among the included studies. The possible sources of heterogeneities among the included studies were also verified using subgroup and sensitivity analysis. The pooled estimates were presented with their 95% CIs. The results of meta-analyses were presented using forest plot, summary tables and texts.

### Publication bias and heterogeneity

Publication bias was checked by looking the asymmetry and confirmed by Egger’s regression test at a 5% significant level [17]. Forest plot, I^2^ test and the Cochrane Q statistics were used to detect heterogeneity among studies [18]. The I^2^ values of 25%, 50%, and 75% were interpreted as low, medium, and high heterogeneity, respectively [19]. In this study, heterogeneity was considered significant when the I^2^ value was ≥ 50%, with p-value < 0.05. Besides, the possible sources of significant heterogeneity were addressed through sensitivity and sub-group analyses.

## Results

### Identification of studies

**Fig. 1:**
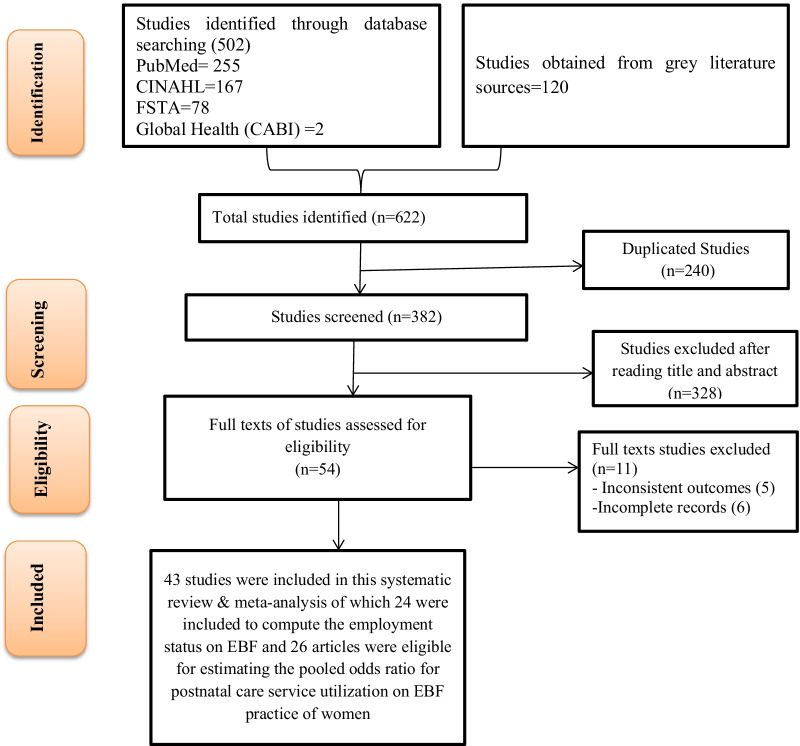
PRISMA flow chart of study selection for a systematic review and meta-analysis of the effects of employment status and postnatal care service utilization on EBF practice of women in Ethiopia

In this systematic review and meta-analysis, 622 articles were identified in the initial search. Of these, 240 were found duplicate articles. A total of 382 articles were reviewed using title and abstract, of which 328 non-important articles were excluded accordingly. Eventually, the full texts of 54 articles were reviewed comprehensively, of which 42 articles [8, 9, 20–59] were found eligible to the current study. Nine articles were excluded after the full text review, due to inconsistent reports [60–64] and incomplete results [64–69]. Of the total articles included to this study, 24 articles were eligible to pooled estimates of the effect of employment status on the EBF practice of women in Ethiopia. Likewise, 26 articles were utilized to estimate the pooled odds ratio indicating the association between post-natal care service utilization on the EBF practice (Fig. 1, Table 1).

**Table 1:** Description of the included studies to identify the effect of employment status and postnatal care on EBF practice

Author year	Study region	Study population (months)	Sample size	EBF practice in number	Factor		EBF		Quality score
							Yes	No	
Gedefaw and Berhe 2015 [1]	Amhara	7–24	144	127	ES	Yes	50	41	6
						No	72	81	
Gebremedhin et al. 2021 [2]	Nationwide	0–23	1406	243	ES	Yes	212	869	8
						No	35	290	
Asemahagn 2016 [3]	Amhara	< 6	332	273	ES	Yes	78	31	8
						No	184	39	
Tewabe et al. 2016 [4]	Amhara	< 6	405	212	ES	Yes	30	52	8
						No	173	130	
Chekol et al. 2017 [5]	Amhara	7–12	649	226	ES	Yes	66	250	8
						No	160	173	
Adugna et al. 2017 [6]	SNNP	< 6	529	322	ES	Yes	114	112	8
						No	208	95	
Tsegaye et al. 2019 [7]	Afar	< 6	618	340	ES	Yes	61	59	8
						No	279	219	
Sinshaw et al. 2015 [8]	Amhara	< 6	803	296	ES	Yes	13	33	5
						No	76	39	
Setegn et al. 2012 [9]	Oromia	< 6	608	434	ES	Yes	3	6	8
						No	196	74	
Berhe et al. 2013 [10]	Tigray	< 6	361	196	ES	Yes	29	45	5
						No	81	26	
Alemayehu et al. 2014 [11]	Tigray	6–12	418	171	ES	Yes	22	32	5
						No	152	212	
Shifraw et al. 2015 [12]	A.A	< 6	648	190	ES	Yes	45	144	6
						No	133	276	
Mekuria and Edris 2015 [13]	Amhara	< 6	413	251	ES	Yes	136	111	8
						No	115	51	
Desalew et al. 2018 [14]	Dire Dawa	6–23	704	571	ES	Yes	360	98	6
						No	211	35	
Elyas et al. 2017 [15]	A.A	< 24	380	168	ES	Yes	69	101	8
						No	99	111	
Ayele 2021 [16]	Amhara	< 24	408	351	ES	Yes	55	86	6
						No	200	67	
Meberatu et al. 2020 [17]	SNNP	< 18	209	171	ES	Yes	65	8	6
						No	104	30	
Bekere et al. 2014 [18]	Oromia	< 6	119	86	ES	Yes	15	7	6
						No	46	12	
Belachew et al. 2018 [19]	Amhara	< 6	472	408	ES	Yes	179	41	8
						No	229	23	
Abera 2012 [20]	Harar	< 24	583	207	ES	Yes	85	104	8
						No	22	88	
Tadesse et al. 2019 [21]	Somalia	3–5	583	397	ES	Yes	28	85	6
						No	369	76	
Tsegaw et al. 2021 [22]	Nationwide	< 6	1185	681	ES	Yes	275	172	8
						No	406	332	
Reddy and Abuka 2014 [23]	Amhara	< 24	347	200	ES	Yes	6	32	6
						No	194	117	
Seid et al. 2013 [24]	Amhara	< 12	819	412	ES	Yes	120	206	6
						No	292	201	
Mamo et al. 2020 [25]	Oromia	6–9	710	464	PNC	Yes	337	104	6
						No	127	142	
Azeze et al. 2019 [26]	SNNP	6–12	403	261	PNC	Yes	187	73	6
						No	74	69	
Hussien et al. 2018 [27]	Afar	< 6	544	–	PNC	Yes	167	78	6
						No	309	22	
Asefaw et al. 2015 [28]	Amhara	< 12	634	435	PNC	Yes	375	149	8
						No	60	50	
Biks et al. 2015 [29]	Amhara	< 6	1769	543	PNC	Yes	197	344	8
						No	346	882	
Asemahagn et al. 2016 [3]	Amhara	< 6	332	273	PNC	Yes	137	25	8
						No	125	45	
Tadesse et al. 2016 [30]	SNNP	0–5	579	305	PNC	Yes	204	127	6
						No	66	117	
Tewabe et al. 2016 [4]	Amhara	< 6	405	212	PNC	Yes	116	81	8
						No	87	121	
Genetu et al. 2017 [31]	Amhara	< 6	367	317	PNC	Yes	304	43	8
						No	13	7	
Ayalew 2020 [32]	Amhara	< 6	400	230	PNC	Yes	238	106	8
						No	43	13	
Sinshaw et al. 2015 [8]	Amhara	< 6	483	296	PNC	Yes	53	31	5
						No	36	41	
Shifraw et al. 2015 [12]	A.A	< 6	648	190	PNC	Yes	154	278	6
						No	32	168	
Mekuria and Edris 2015 [13]	Amhara	< 6	413	251	PNC	Yes	199	79	8
						No	52	83	
Fufa et al. 2021 [33]	Oromia	< 6	542	384	PNC	Yes	221	117	6
						No	179	48	
Beyene et al. 2019 [34]	Afar	< 6	465	301	PNC	Yes	181	87	6
						No	119	78	
Hunegnaw et al. 2017 [35]	Amhara	< 12	478	354	PNC	Yes	290	75	8
						No	68	45	
Hoche et al. 2017 [36]	SNNP	6–12	634	549	PNC	Yes	181	19	8
						No	368	66	
Arage and Gedamu 2016 [37]	Amhara	6–12	453/470	321	PNC	Yes	251	73	6
						No	83	46	
Ayele 2021 [16]	Amhara	< 24	408	351	PNC	Yes	227	85	6
						No	28	68	
Meberatu et al. 2020 [17]	SNNP	< 18	209	207	PNC	Yes	151	27	6
						No	18	11	
Kelkay et al. 2020 [38]	Amhara	6–9	344	187	PNC	Yes	192	37	6
						No	78	37	
Sefene et al. 2013 [39]	Amhara	< 6	159	78	PNC	Yes	27	19	5
						No	51	62	
Tsegaw et al. 2021 [22]	Nationwide	< 6	1185	555	PNC	Yes	35	39	8
						No	646	465	
Lenja et al. 2016 [40]	SNNP	< 6	396	309	PNC	Yes	188	33	8
						No	121	54	
Kebede et al. 2020 [41]	Oromia	6–24	313	237	PNC	Yes	73	30	8
						No	164	46	
Hagos and Tadesse 2020 [42]	SNNP	< 6	584	514	PNC	Yes	308	32	8
						No	204	37	

SNNP: Southern Nations Nationalities and Peoples; AA: Addis Ababa; PNC: Post-natal Care; ES: Employment Status; EBF: Exclusive Breast Feeding

### Description included studies

A total of 23,224 study populations were included in this systematic review and meta-analysis. The included articles were published from 2012 to 2021 and most of the studies were conducted in Amhara region (19 studies) followed by SNNP where seven studies were conducted in. The rest were conducted in Oromia (five studies), Afar (three studies), Tigray (two studies), Addis Ababa (two studies), Harari (one study), Diredawa (one study), Somali regions (one study), and nationwide (two studies). Regarding the sample size of the included studies, it ranged from 119 [28] to 1769 [32] in which both these studies were from Amhara region. The prevalence of EBF practice among the individual studies also ranged from 17.28% [37] to 88.19% [38]. Regarding quality scores of the studies, the JBI checklist of critical appraisal of observational studies was used. Thus, 21 articles were classified under high quality and other 21 articles were classified under medium quality.

### The effect of employment status on exclusive breast-feeding practice of women in Ethiopia

**Fig. 2:**
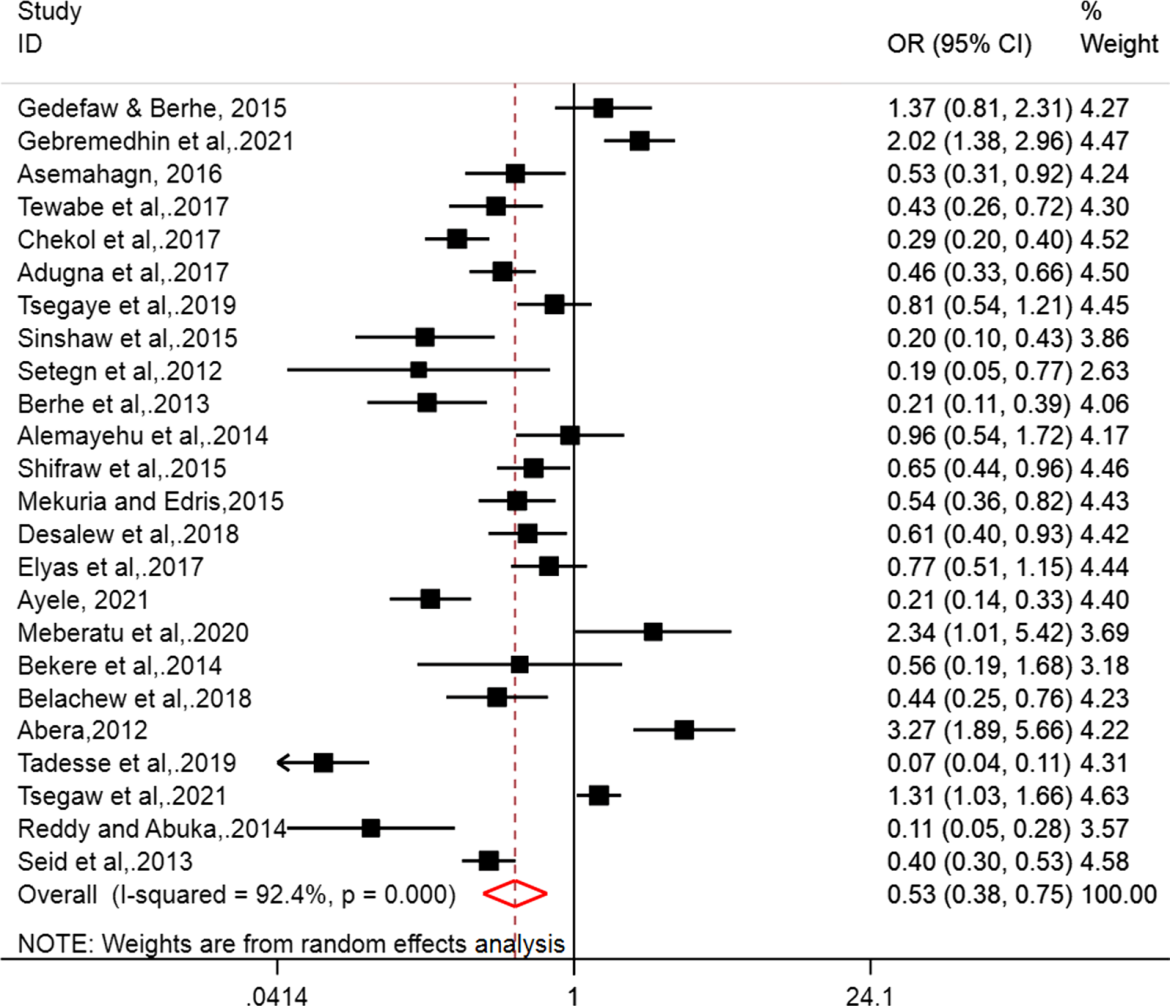
Forest plot of the effect of employment status on the EBF practice of women in Ethiopia

The association between employment statuses of women with EBF practice of women was assessed by computing the pooled odds ratio. A total of 24 articles were eligible to estimate the POR [8, 9, 20–22, 24, 28–30, 33–35, 37, 38, 48–51, 53–55, 57–59]. Thus, employment status of women was significantly associated with the EBF practice of women in Ethiopia. Unemployed women were 47% more likely to practice EBF than the counterpart employed women in Ethiopia (POR = 0.53, 95% CI 0.38, 0.78). The POR was computed using random effect model due the presence of significant heterogeneity among the included studies (I^2^ = 92.4%, p < 0.001) (Fig. 2).

**Fig. 3:**
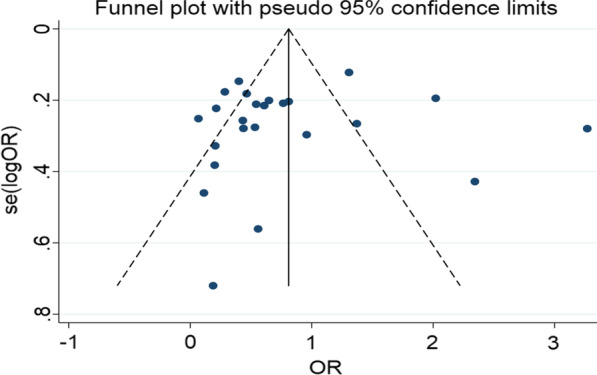
Funnel plot of the effect of employment status on the EBF practice of women in Ethiopia

The possible sources of higher heterogeneity were further explored using funnel plot and Egger’s regression test. The funnel plot was found to be symmetrical and it was objectively confirmed by the Egger’s regression test in that it was statistically insignificant (intercept [B0] = 0.908, 95% CI 0.070, 1.744) and the p-value of bias was 0.801 (Fig. 3).

**Fig. 4:**
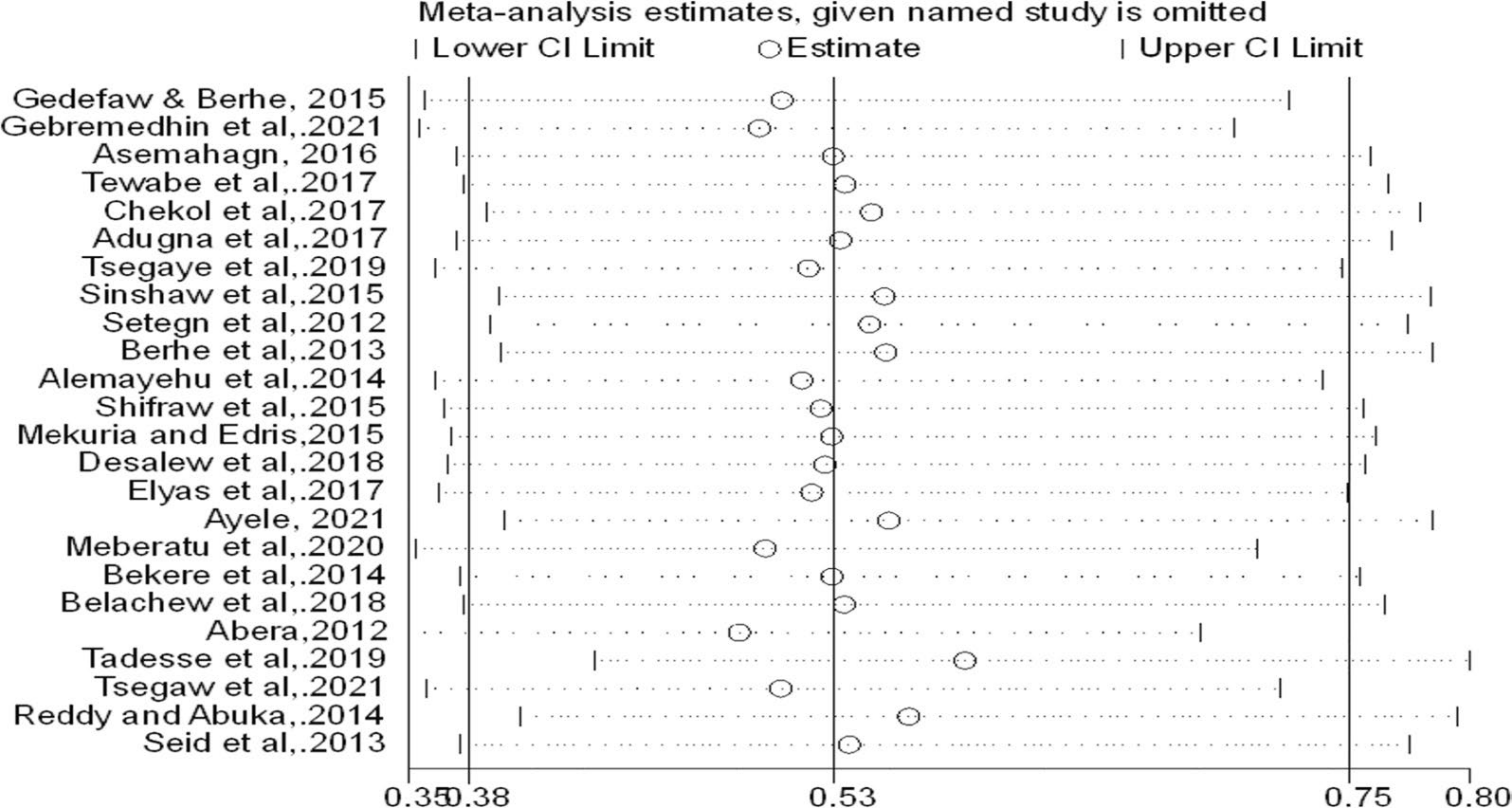
Sensitivity analysis of the effect of employment status on the EBF practice of women in Ethiopia

Furthermore, subgroup and sensitivity analyses were performed to explore the possible sources of heterogeneity. However, heterogeneity remained high among the subgroup estimates. The sensitivity analysis also implied that there is no single study responsible to this remarkable heterogeneity (Fig. 4).

**Fig. 5:**
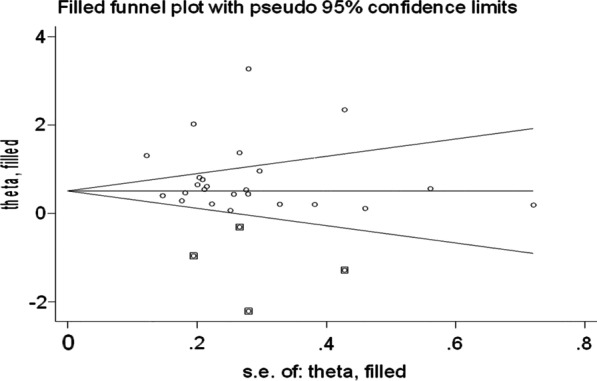
Funnel plot based on trim fill analysis of the effect of employment status on the EBF practice of women in Ethiopia

Eventually, the trim fill analysis was done to find out the source of heterogeneity and it was found that four studies that were imputed could be the source of heterogeneity. Thus, POR was found to be 0.51 (95% CI 0.16, 0.86). The funnel plot was also computed based on trim fill analysis (Fig. 5).

### The effect of post-natal care service utilization on EBF practice of women in Ethiopia

**Fig. 6:**
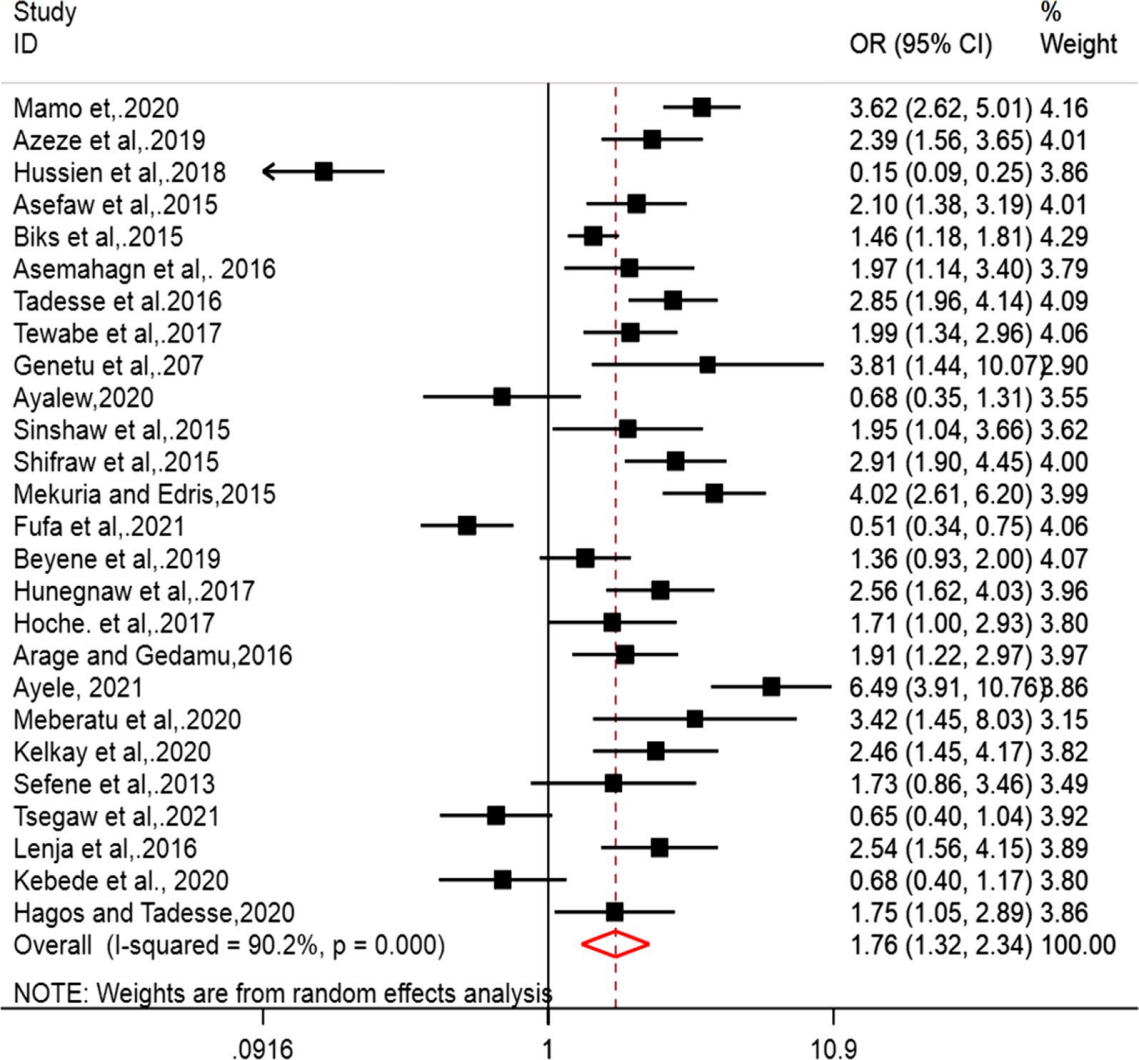
Forest plot of the effect of postnatal care service utilization on EBF practice of women in Ethiopia

The effect of postnatal service utilization on the EBF practice of women was determined using 26 articles [23–27, 31, 32, 36, 39–50, 52–54, 56–58]. Post-natal care service utilization was significantly associated with the EBF practice of women in Ethiopia. Women those who attend post-natal care were 1.76 times more likely to practice EBF as compared to women who did not attend postnatal care (POR = 1.76, 95% CI 1.32, 2.34). The POR was estimated using random effect model due higher heterogeneity among the studies included to this meta-analysis (I^2^ = 90.2%, p < 0.001) (Fig. 6).

**Fig. 7:**
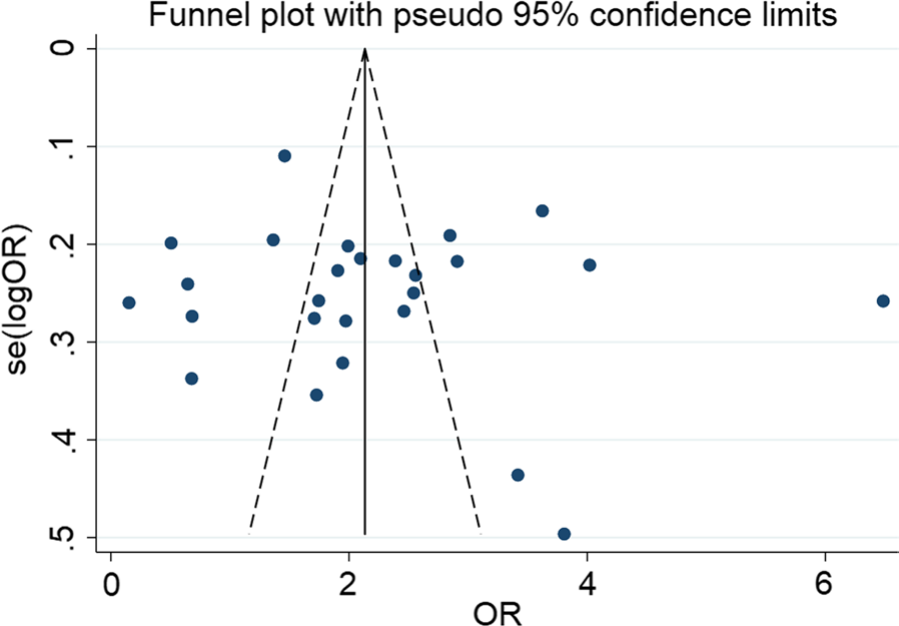
Funnel plot of the effect of postnatal care service utilization on EBF practice of women in Ethiopia

The possible sources of higher heterogeneity were computed using different parameters. Primarily, funnel plot was drawn to identify the effect of small studies as a source of bias. However, the funnel plot looked symmetrical and the asymmetry was confirmed objectively using egger’s regression test (Bo = 1.681, 95% CI − 0.023, 3.387) with a p-value of bias of 0.572 (Fig. 7).

**Fig. 8:**
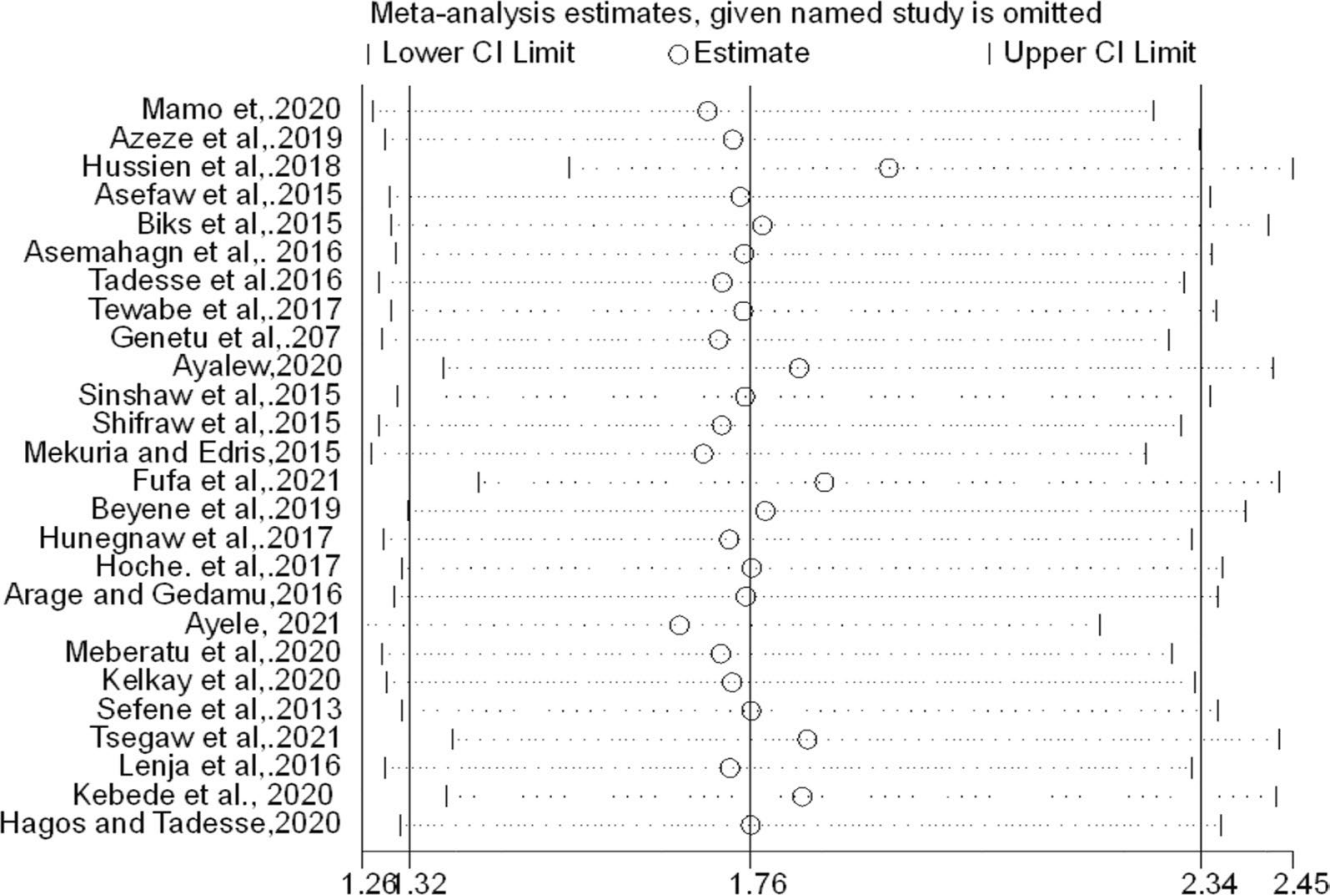
Funnel plot of the effect of postnatal care service utilization on EBF practice of women in Ethiopia

Secondly, sensitivity analysis was performed to identify the specific studies that might contribute to the higher heterogeneity in the pooled estimate of the current meta-analysis. However, the graph indicated that all studies could have contribution to the presence of significant heterogeneity (Fig. 8).

Finally, the trim-fill analysis was employed to identify the effects of studies that are not reported and that could have effect on the pooled estimate as well as source of higher heterogeneity. Despite this, the analysis indicated that there were no small studies that could modify the POR and the POR was kept 1.76 after trim-fill analysis. Furthermore, univariate Meta regression was performed considering differences in the sample size as a possible source of higher heterogeneity. But, the result depicted that sample size was not the source of heterogeneity (coefficient = − 0.0009488, 95% CI − 0.0026782, 0.0007806, p = 0.269).

### The pooled prevalence of EBF of practice in Ethiopia

**Fig. 9:**
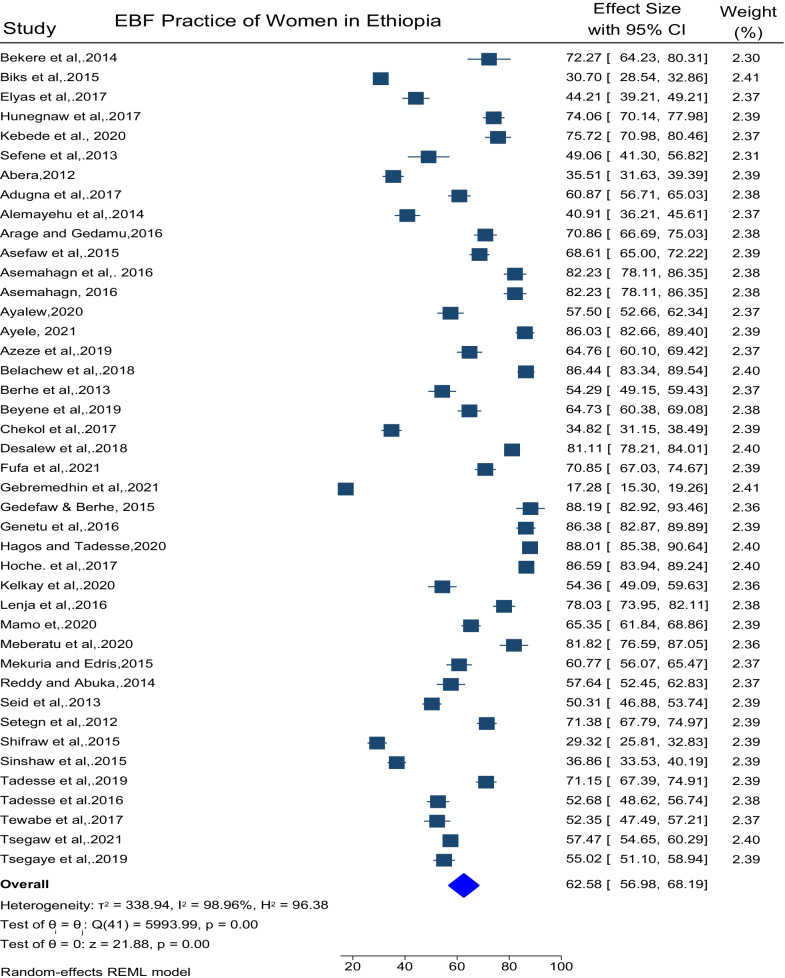
Pooled prevalence of EBF in Ethiopia

The prevalence EBF in Ethiopia was also computed using 42 eligible articles and it was found that 62.58% (95% CI 56.98, 68.19, I^2^ = 96.4%, p < 0.001) of women found practicing EBF (Fig. 9).

## Discussion

In the current systematic review and meta-analysis, the effects of employment status of women and post-natal care service utilization on the exclusive breastfeeding practice of women in Ethiopia were determined. Thus, unemployed women were found to have good EBF practice as compared to the employed ones. Likewise, women who had history of post-natal care service utilization practiced EBF more likely than women who do not attend post-natal care. The pooled prevalence of EBF practice was also computed from the included studies.

Unemployed women were 49% more likely to practice EBF than women who are employed in different governmental institutions. Different mechanisms could explain the observed association between unemployed women and EBF practice. Employed mothers face more challenge in physical and emotional disturbance to continue EBF practice. Evidence suggested that being employed and working at different organizations has been one of the greatest barriers to EBF practice [33, 55, 70, 71]. Our study finding is also in line with meta-analysis result of observational studies where full time maternal employment was negatively associated with the practice of EBF in comparison to unemployed mothers. Those with full time employment in the first 6 months were 57% less likely to practice EBF [72]. The same is true for a community based cross sectional study in Ghana, being employed significantly reduce the practice of EBF to 36% with (p = 0.000) [73]. A meta-analysis done throughout the world on the overall prevalence of exclusive breastfeeding among employed mother also showed this fact. Being employed significantly reduce exclusive breastfeeding practice 25% (95% CI 21% to 29%) [74].

Similarly, in this review, the likelihood of EBF practice among women who attend post-natal care is 1.76 times as compared to women who do not attend post-natal care**.** Several researches revealed that post-natal counseling components were more effective than counseling on antenatal; where mothers encounter most challenges in the early period after birth, where they are developing breastfeeding skills [75]. Post-natal care counseling emphasis on the dyadic interaction between a healthcare worker and a mother, rather than the top-down education-based approach [75]. An additional possible explanation for the increased effect of post-natal counseling on EBF practice might be its contribution for the actual and potential difficulties to work with women on ways to overcome the challenges during EBF practice [76].

Lastly, the pooled prevalence of EBF is computed and 62.58% of women practice EBF. The overall prevalence of EBF in this study is similar to the result of a meta-analysis conducted in Ethiopia (60.42%) [72].The pooled prevalence of EBF in this review is also higher than the study done in sub Saharan countries (36%) [77], Central Africa (23.70%) and Southern Africa (5.57%) [78]. This variation could be due infant and maternal socio-demographic variations, health service utilization, the number of studies used in and methodological difference.

In general, this review found that, the practice of EBF will be more likely, for mothers with better access for post natal counseling. Recent programmatic guide lines from the WHO and UNICEF outline the need to improve newborn feeding and points to post natal care promotion and support as a potential mechanism to increase EBF practice [79]. In a meta-analysis of 15 sub-Saharan African countries on the role of PNC in improving newborn feeding, PNC were more likely to increase EBF practice among 11 of 15 countries [80]. Exclusive breastfeeding practice is one of the most effective means to reduce under nutrition, an underlying cause of under-five mortality and may also have longer term health benefits for the mother [81]. The review on the effects of maternal employment status demonstrated that maternal employment status significantly decreases the practice of EBF. Maternal employment affects child caring time and is reported to be the major reason for low rate and duration of EBF [10]. Empowering women to exclusively breastfeed with different governmental and non-governmental organization should be encouraged. By enacting 6 months mandatory paid maternity leave can increase the rate of exclusive breastfeeding in the first 6 months of life up to 50% [72].

The maximum effort to find out studies from multiple data bases and grey literatures as well as the extensive analyses performed to compute pooled estimates were the strength of the study. However, including studies only in English may cause either under or over estimation of the meta-analyses findings.

## Conclusion

The current study found that utilization of post-natal care service and being unemployed were found to enhance the breast feeding practice of women in Ethiopia. Thus, promoting the post-natal care service, the most underachieved health care service indicator in Ethiopia of whom only 34% [82] women attend post-natal care in Ethiopia, could be the best mechanism to enhance EBF practice. Like-wise, extending the maternal leave period up to 6 months after birth for women working in governmental institutions could improve the EBF practice in Ethiopia.

## Supplementary Information

**Additional file 1. **PRISMA 2009 checklist.

**Additional file 2.** Critical appraisal of cross sectional studies.

## Data Availability

The data that support the review findings of this study are included in the manuscript and with supporting files.

## Abbreviations

EBF: Exclusive breast feeding
FSTA: Food Science and Technology Abstracts
JBI: Juana Brigg’s Institute
POR: Pooled odds ratio
PRISMA: Preferred Reporting Items for Systematic Reviews and Meta-analysis
SDG: Sustainable Development Goal
SNNP: Southern Nations Nationalities and Peoples
