# Supplementary material for: The effect of unemployment and post-natal care on the exclusive breast-feeding practice of women in Ethiopia: a systematic review and meta-analysis
Source: Reprod Health. 2022 Apr 15;19:94. doi: 10.1186/s12978-022-01404-y (PMC9013047; doi:10.1186/s12978-022-01404-y)
Supplement: Supplementary file 2 — Additional file 2. Critical appraisal of cross sectional studies. [file 12978_2022_1404_MOESM2_ESM.docx]

**Critical appraisal of cross sectional studies**

| Author, year | Q1 | | | | Q2 | | | | Q3 | | | | | Q4 | | | | | Q5 | | | | Q6 | | | | Q7 | | | | Q8 | | | | Overall quality result |
| --- | --- | --- | --- | --- | --- | --- | --- | --- | --- | --- | --- | --- | --- | --- | --- | --- | --- | --- | --- | --- | --- | --- | --- | --- | --- | --- | --- | --- | --- | --- | --- | --- | --- | --- | --- |
|  | Y | N | U | NA | Y | N | U | NA | | Y | N | U | NA | | Y | N | U | NA | Y | N | U | NA | Y | N | U | NA | Y | N | U | NA | Y | N | U | NA |  |
| Bekere et al,.2014 | √ |  |  |  | √ |  |  |  | | √ |  |  |  | | √ |  |  |  |  |  |  | √ |  |  |  | √ | √ |  |  |  | √ |  |  |  | 6/8(75%) |
| Biks et al,.2015 | √ |  |  |  | √ |  |  |  | | √ |  |  |  | | √ |  |  |  | √ |  |  |  | √ |  |  |  | √ |  |  |  | √ |  |  |  | 8/8(100%) |
| Elyas et al,.2017 | √ |  |  |  | √ |  |  |  | | √ |  |  |  | | √ |  |  |  | √ |  |  |  | √ |  |  |  | √ |  |  |  | √ |  |  |  | 8/8(100%) |
| Hunegnaw et al,.2017 | √ |  |  |  | √ |  |  |  | | √ |  |  |  | | √ |  |  |  | √ |  |  |  | √ |  |  |  | √ |  |  |  | √ |  |  |  | 8/8(100%) |
| Kebede et al., 2020 | √ |  |  |  | √ |  |  |  | | √ |  |  |  | | √ |  |  |  | √ |  |  |  | √ |  |  |  | √ |  |  |  | √ |  |  |  | 8/8(100%) |
| Sefene et al,.2013 | √ |  |  |  | √ |  |  |  | | √ |  |  |  | | √ |  |  |  | √ |  |  |  | √ |  |  |  | √ |  |  |  | √ |  |  |  | 8/8(100%) |
| Abera,2012 | √ |  |  |  | √ |  |  |  | | √ |  |  |  | | √ |  |  |  | √ |  |  |  | √ |  |  |  | √ |  |  |  | √ |  |  |  | 8/8(100%) |
| Adugna et al,.2017 | √ |  |  |  | √ |  |  |  | | √ |  |  |  | | √ |  |  |  | √ |  |  |  | √ |  |  |  | √ |  |  |  | √ |  |  |  | 8/8(100%) |
| Alemayehu et al,.2014 | √ |  |  |  | √ |  |  |  | | √ |  |  |  | |  |  | √ |  |  |  |  | √ |  |  |  | √ | √ |  |  |  | √ |  |  |  | 5/8(62.5%) |
| Arage and Gedamu,2016 | √ |  |  |  | √ |  |  |  | | √ |  |  |  | | √ |  |  |  |  |  |  | √ |  |  |  | √ | √ |  |  |  | √ |  |  |  | 6/8(75%) |
| Asefaw et al,.2015 | √ |  |  |  | √ |  |  |  | | √ |  |  |  | | √ |  |  |  | √ |  |  |  | √ |  |  |  | √ |  |  |  | √ |  |  |  | 8/8(100%) |
| Asemahagn et al,. 2016 | √ |  |  |  | √ |  |  |  | | √ |  |  |  | | √ |  |  |  | √ |  |  |  | √ |  |  |  | √ |  |  |  | √ |  |  |  | 8/8(100%) |
| Asemahagn, 2016 | √ |  |  |  | √ |  |  |  | | √ |  |  |  | | √ |  |  |  | √ |  |  |  | √ |  |  |  | √ |  |  |  | √ |  |  |  | 8/8(100%) |
| Ayalew,2020 | √ |  |  |  | √ |  |  |  | | √ |  |  |  | | √ |  |  |  | √ |  |  |  | √ |  |  |  | √ |  |  |  | √ |  |  |  | 8/8(100%) |
| Ayele, 2021 | √ |  |  |  | √ |  |  |  | | √ |  |  |  | | √ |  |  |  |  |  |  | √ |  |  |  | √ | √ |  |  |  | √ |  |  |  | 6/8(75%) |
| Azeze et al,.2019 | √ |  |  |  | √ |  |  |  | | √ |  |  |  | | √ |  |  |  |  |  |  | √ |  |  |  | √ | √ |  |  |  | √ |  |  |  | 6/8(75%) |
| Belachew et al,.2018 | √ |  |  |  | √ |  |  |  | | √ |  |  |  | | √ |  |  |  | √ |  |  |  | √ |  |  |  | √ |  |  |  | √ |  |  |  | 8/8(100%) |
| Berhe et al,.2013 | √ |  |  |  | √ |  |  |  | | √ |  |  |  | |  |  | √ |  |  |  |  | √ |  |  |  | √ | √ |  |  |  | √ |  |  |  | 5/8(62.5%) |
| Beyene et al,.2019 | √ |  |  |  | √ |  |  |  | | √ |  |  |  | | √ |  |  |  |  |  |  | √ |  |  |  | √ | √ |  |  |  | √ |  |  |  | 6/8(75%) |
| Chekol et al,.2017 | √ |  |  |  | √ |  |  |  | | √ |  |  |  | | √ |  |  |  | √ |  |  |  | √ |  |  |  | √ |  |  |  | √ |  |  |  | 8/8(100%) |
| Desalew et al,.2018 | √ |  |  |  | √ |  |  |  | | √ |  |  |  | | √ |  |  |  |  |  |  | √ |  |  |  | √ | √ |  |  |  | √ |  |  |  | 6/8(75%) |
| Fufa et al,.2021 | √ |  |  |  | √ |  |  |  | | √ |  |  |  | | √ |  |  |  |  |  |  | √ |  |  |  | √ | √ |  |  |  | √ |  |  |  | 6/8(75%) |
| Gebremedhin et al,.2021 | √ |  |  |  | √ |  |  |  | | √ |  |  |  | | √ |  |  |  | √ |  |  |  | √ |  |  |  | √ |  |  |  | √ |  |  |  | 8/8(100%) |
| Gedefaw & Berhe, 2015 | √ |  |  |  | √ |  |  |  | | √ |  |  |  | | √ |  |  |  |  |  |  | √ |  |  |  | √ | √ |  |  |  | √ |  |  |  | 6/8(75%) |
| Genetu et al,.2016 | √ |  |  |  | √ |  |  |  | | √ |  |  |  | | √ |  |  |  | √ |  |  |  | √ |  |  |  | √ |  |  |  | √ |  |  |  | 8/8(100%) |
| Hagos and Tadesse,2020 | √ |  |  |  | √ |  |  |  | | √ |  |  |  | | √ |  |  |  | √ |  |  |  | √ |  |  |  | √ |  |  |  | √ |  |  |  | 8/8(100%) |
| Hoche. et al,.2017 | √ |  |  |  | √ |  |  |  | | √ |  |  |  | | √ |  |  |  | √ |  |  |  | √ |  |  |  | √ |  |  |  | √ |  |  |  | 8/8(100%) |
| Hussien et al,.2018 | √ |  |  |  | √ |  |  |  | | √ |  |  |  | | √ |  |  |  |  |  |  | √ |  |  |  | √ | √ |  |  |  | √ |  |  |  | 6/8(75%) |
| Kelkay et al,.2020 | √ |  |  |  | √ |  |  |  | | √ |  |  |  | | √ |  |  |  |  |  |  | √ |  |  |  | √ | √ |  |  |  | √ |  |  |  | 6/8(75%) |
| Lenja et al,.2016 | √ |  |  |  | √ |  |  |  | | √ |  |  |  | | √ |  |  |  | √ |  |  |  | √ |  |  |  | √ |  |  |  | √ |  |  |  | 8/8(100%) |
| Mamo et,.2020 | √ |  |  |  | √ |  |  |  | | √ |  |  |  | | √ |  |  |  |  |  |  | √ |  |  |  | √ | √ |  |  |  | √ |  |  |  | 6/8(75%) |
| Meberatu et al,.2020 | √ |  |  |  | √ |  |  |  | | √ |  |  |  | | √ |  |  |  |  |  |  | √ |  |  |  | √ | √ |  |  |  | √ |  |  |  | 6/8(75%) |
| Mekuria and Edris,2015 | √ |  |  |  | √ |  |  |  | | √ |  |  |  | | √ |  |  |  | √ |  |  |  | √ |  |  |  | √ |  |  |  | √ |  |  |  | 8/8(100%) |
| Reddy and Abuka,.2014 | √ |  |  |  | √ |  |  |  | | √ |  |  |  | | √ |  |  |  |  |  |  | √ |  |  |  | √ | √ |  |  |  | √ |  |  |  | 6/8(75%) |
| Seid et al,.2013 | √ |  |  |  | √ |  |  |  | | √ |  |  |  | | √ |  |  |  |  |  |  | √ |  |  |  | √ | √ |  |  |  | √ |  |  |  | 6/8(75%) |
| Setegn et al,.2012 | √ |  |  |  | √ |  |  |  | | √ |  |  |  | | √ |  |  |  | √ |  |  |  | √ |  |  |  | √ |  |  |  | √ |  |  |  | 8/8(100%) |
| Shifraw et al,.2015 | √ |  |  |  | √ |  |  |  | | √ |  |  |  | | √ |  |  |  |  |  |  | √ |  |  |  | √ | √ |  |  |  | √ |  |  |  | 6/8(75%) |
| Sinshaw et al,.2015 | √ |  |  |  | √ |  |  |  | | √ |  |  |  | |  |  | √ |  |  |  |  | √ |  |  |  | √ | √ |  |  |  | √ |  |  |  | 5/8(62.5%) |
| Tadesse et al,.2019 | √ |  |  |  | √ |  |  |  | | √ |  |  |  | | √ |  |  |  |  |  |  | √ |  |  |  | √ | √ |  |  |  | √ |  |  |  | 6/8(75%) |
| Tadesse et al.2016 | √ |  |  |  | √ |  |  |  | | √ |  |  |  | | √ |  |  |  |  |  |  | √ |  |  |  | √ | √ |  |  |  | √ |  |  |  | 6/8(75%) |
| Tewabe et al,.2017 | √ |  |  |  | √ |  |  |  | | √ |  |  |  | | √ |  |  |  | √ |  |  |  | √ |  |  |  | √ |  |  |  | √ |  |  |  | 8/8(100%) |
| Tsegaw et al,.2021 | √ |  |  |  | √ |  |  |  | | √ |  |  |  | | √ |  |  |  | √ |  |  |  | √ |  |  |  | √ |  |  |  | √ |  |  |  | 8/8(100%) |
| Tsegaye et al,.2019 | √ |  |  |  | √ |  |  |  | | √ |  |  |  | | √ |  |  |  | √ |  |  |  | √ |  |  |  | √ |  |  |  | √ |  |  |  | 8/8(100%) |
| Sefene et al,.2013 | √ |  |  |  | √ |  |  |  | | √ |  |  |  | |  |  | √ |  |  |  |  | √ |  |  |  | √ | √ |  |  |  | √ |  |  |  | 5/8(62.5%) |

****Y=yes, N=no, U=unclear, NA=not applicable, <60%=low, 60-80%=medium, >80%=high quality***
